# Eco-Bio-Social Determinants for House Infestation by Non-domiciliated *Triatoma dimidiata* in the Yucatan Peninsula, Mexico

**DOI:** 10.1371/journal.pntd.0002466

**Published:** 2013-09-26

**Authors:** Eric Dumonteil, Pierre Nouvellet, Kathryn Rosecrans, Maria Jesus Ramirez-Sierra, Rubi Gamboa-León, Vladimir Cruz-Chan, Miguel Rosado-Vallado, Sébastien Gourbière

**Affiliations:** 1 Laboratorio de Parasitología, Centro de Investigaciones Regionales “Dr. Hideyo Noguchi”, Universidad Autónoma de Yucatán, Mérida, Yucatán, Mexico; 2 Department of Tropical Medicine, School of Public Health and Tropical Medicine, Tulane University, New Orleans, Louisiana, United States of America; 3 UMR 5244 CNRS-UPVD ‘Ecologie et Evolution des Interactions’, Université de Perpignan Via Domitia, Perpignan, France; 4 Medical Research Council Centre for Outbreak Analysis and Modelling, Department of Infectious Disease Epidemiology, Imperial College London, London, United Kingdom; Universidad de Buenos Aires, Argentina

## Abstract

**Background:**

Chagas disease is a vector-borne disease of major importance in the Americas. Disease prevention is mostly limited to vector control. Integrated interventions targeting ecological, biological and social determinants of vector-borne diseases are increasingly used for improved control.

**Methodology/principal findings:**

We investigated key factors associated with transient house infestation by *T. dimidiata* in rural villages in Yucatan, Mexico, using a mixed modeling approach based on initial null-hypothesis testing followed by multimodel inference and averaging on data from 308 houses from three villages. We found that the presence of dogs, chickens and potential refuges, such as rock piles, in the peridomicile as well as the proximity of houses to vegetation at the periphery of the village and to public light sources are major risk factors for infestation. These factors explain most of the intra-village variations in infestation.

**Conclusions/significance:**

These results underline a process of infestation distinct from that of domiciliated triatomines and may be used for risk stratification of houses for both vector surveillance and control. Combined integrated vector interventions, informed by an Ecohealth perspective, should aim at targeting several of these factors to effectively reduce infestation and provide sustainable vector control.

## Author Summary

Chagas disease is a parasitic disease of major importance in the Americas, transmitted by triatomine insects. Integrated control interventions targeting a combination of factors associated with the presence of the insect vectors are increasingly investigated for improved control. Here we identified the factors associated with the seasonal intrusion of triatomine vectors in houses from the Yucatan peninsula, Mexico, by studying the characteristics of 308 houses from 3 villages. The presence of triatomine vectors was associated with the presence of dogs, chickens and potential bug refuges, such as rock piles, and the proximity of houses to vegetation at the periphery of the villages and to public light sources. Thus, factors favoring seasonal intrusion of triatomines appear different from those favoring their domiciliation. Integrated control interventions based on this Ecohealth perspective should focus on several of the factors identified in this study to achieve effective and sustainable vector control.

## Introduction

Chagas disease is a vector-borne disease of major importance in the Americas where it is endemic. It affects an estimated 8–10 million people, and nearly 25 millions are at risk of infection [1]. In terms of disability-adjusted life years (DALYs), and with a burden of 0.7 million DALYs, it is the fourth most important disease in Latin America, following only hookworm, ascaris and trichuris infections [2]. The disease is caused by the protozoan parasite *Trypanosoma cruzi* and most transmission occurs by hematophagous triatomine vectors [3].

Disease control and prevention are mostly limited to vector control to reduce triatomine infestation of human dwellings and concomitant transmission of *T. cruzi* to humans [4]. Indoor residual spraying of pyrethroid insecticides and housing improvement are the main methods of interventions. Inter-governmental vector control initiatives during the 1990s are believed to have eliminated vectorial transmission to humans in several Latin American regions [4]. However, this success is mitigated by difficulties in sustaining vector control activities [5] and the emergence of insecticide resistance [6]. Additionally, some triatomine species are not well domiciliated but transiently invade house to feed on humans, representing an important source of infection. However, traditional control methods are less effective at preventing these triatomines from entering houses [3].

Integrated vector control based “on a rational decision-making process for the optimal use of resources in the management of vector populations” [7], targets ecological, biological and social determinants of vector-borne diseases to achieve improved control. It shares a common Ecohealth perspective, an ecosystem approach to health that promotes use of transdisciplinary participatory research to attain better health outcomes through environmental management [8]. Such multidisciplinary strategies are emerging as more rational, sustainable, and cost-effective than widespread empirical insecticide spraying [7], [8]. However, they require extensive knowledge of the eco-bio-social determinants leading to disease transmission.

Most research on Chagas disease vectors has focused on domiciliated *Triatoma infestans*, the major vector species in South America. Risk factors for house infestation (and colonization) by *T. infestans* are typically associated with housing structure and quality; houses providing abundant hiding refuges for bug resting and reproduction (cracked adobe walls, dirt floors, thatched roofs, poor hygiene, darkness, etc..) and easily accessible feeding sources (indoor dogs or chickens, large families) are more likely to be infested [9]–[11]. Similar results have been observed for other domiciliated vector species and populations, including *T. dimidiata* in Central America [12]–[16]. Accordingly, integrated vector control interventions targeting these risk factors such cement floors or roofs, or wall plastering are being evaluated [17]–[19] as alternatives to conventional insecticide spraying [20], [21].

Less is known about determinants of invasion by non-domiciliated triatomines, which have limited ability to establish domestic colonies, but transiently enter houses for blood feeding on animal and human hosts. In urban areas, infestation by *T. pallidipennis* or *T. dimidiata* has been found to be much less dependent on housing characteristics, but is instead associated with the availability of various peridomestic refuges (large peridomestic area, adjacent empty or abandoned lots) and feeding sources such as dogs, squirrels, and opossums [22], [23].

In the Yucatan peninsula, sibling species from the *T. dimidiata* complex typically infest houses on a seasonal basis during the months of March–July, with very limited ability to colonize houses [24]–[27]. This contrasts with its level of domiciliation in Central America and has been hypothesized to be associated with genetic differences within the *T. dimidiata* complex [20], [28]. This infestation is responsible for a seroprevalence of *T. cruzi* infection in humans of about 1–5% in the region [29], [30] and these invasive vectors cannot be fully controlled by conventional insecticide spraying [31], [32]. While we have now an accurate description of the dynamics of house infestation [33], [34], we still have limited understanding of the factors driving this process, limiting the design of vector control interventions. For example, houses located in the periphery of rural villages, in close proximity to the surrounding vegetation, were found twice as likely to be infested compared with houses located closer to village centers [35], suggesting that specific spatial targeting of vector control may be appropriate [36]. Proximity of public street lights was also found to be a significant contributor to infestation [37]. Importantly, housing quality seems irrelevant for this transient infestation [24], but more detailed ecosystemic and social studies are needed to fully identify and understand the interplay of factors contributing to this infestation pattern and to develop integrated vector control interventions.

In the present study, we performed a detailed analysis of the eco-bio-social characteristics of rural villages in the Yucatan peninsula, Mexico, to identify the key determinants associated with transient house infestation by *T. dimidiata*.

## Materials and Methods

### Study sites and socio-cultural context

The study was carried out from July 2010 to July 2011 in the rural villages of Bokoba (21.01°N, 89.07°W), Teya (21.05°N, 89.07°W) and Sudzal (20.87°N, 88.98°W), located about 15–20 km apart in the central part of the Yucatan state, Mexico. The regional climate is warm and humid, with an average annual temperature of 26°C and 1150 mm of rainfall, and the villages are surrounded by a mixture of secondary bush vegetation and agricultural/pasture land. There are a total of 570, 702 and 416 houses in Bokoba, Teya and Sudzal, respectively, all of which have been georeferenced previously [35]. The respective populations are of about 2,000 inhabitants in both Bokoba and Teya and 1,600 in Sudzal, with about 40% of the population below 14 years of age. Most of the population (over 90%) is of Mayan descent and culture. Each of these communities has a health center run by the state public health system (*Secretaria de Salud de Yucatán*), as well as public primary and secondary schools and at least one church. The population is largely Catholic (over 95%). Previous work indicated that there was no significant difference in the overall housing characteristics and living conditions of these villages, all three of them being rather representative of the conditions in rural Yucatan [32]. There has been no systematic vector control program in these villages, but entomologic monitoring by community participation has been performed since 2006 and pilot interventions were implemented in a limited number of houses in 2007 [32].

### Household survey

An extensive survey was developed and validated to identify the key eco-bio-social dimensions of the households. The survey included a total of 127 variables describing in detail housing structure and characteristics (34 variables), peridomicile structure and characteristics (56 variables), socio-demographic characteristics and cultural practices (38 variables) (see Table S1 for the complete list of those variables).

Housing structure variables included the number of rooms and construction materials of the different parts of the house (floor, wall, roof). Peridomicile variables included data on the size of the peridomicile, its vegetation, the presence of different structures (storage, corrals, others) and the presence of different species of domestic animals. Socio-demographic characteristics and cultural practices included a detailed description of the composition of the household, its socioeconomic status, and common practices related to the maintenance and care of the house and the peridomicile, including use of insecticides and other potential vector control measures (e.g. storage of grains or construction material, cleaning habits, etc…). Practices related to the care of domestic animals were also investigated.

A total of 346 households randomly sampled in the three villages was selected for the survey (representing 20% of the total number of households). We used a random sampling scheme to avoid any bias in selecting specific households and ensure that all types of households would be included in the survey, irrespective of the housing type, structure, position in the village, infestation status. Teams of two trained field workers performed individual visits to each household to apply the survey following obtention of written informed consent. The protocol was approved by both the World Health Organization and the Autonomous University of Yucatan institutional bioethics committees. We were able to obtain data from 308 households, the remaining being abandoned houses, households that declined to participate, or households in which inhabitants were unavailable after three visits.

### House infestation

In all three villages, house infestation was monitored from July 2010 to July 2011 by community participation, which we have found highly reliable and more sensitive than timed manual searches for entomologic surveys of houses with low and transient infestation [24], [25]. Infestation was defined as the catch and notification of at least one triatomine (adult or nymph) inside the domicile at any time of the year. Community members were asked to collect any triatomine-like bug observed in their houses, using plastic bags to avoid direct contact with the bugs, and take them to the health center where the bugs were registered together with basic information on the household. We later visited each household that had collected a bug to confirm the coordinates we had previously georeferenced [35]. Regular community meetings were held during the study to promote Chagas disease awareness and ensure community participation. Conventional indices were calculated to describe infestation: infestation index (percent of houses with indoor triatomine presence at any time of the year), colonization index (percent of infested houses with nymphal stages), density index (number of triatomines per infested house) [23], [24].

### Univariate and multivariate analysis

We used a mixed modeling approach by first performing univariate analyses of the 127 variables describing the eco-bio-social conditions of the 308 houses using logistic regression, to explore the potential association between the transient presence of *T. dimidiata* at any time of the year (house infestation) and these variables. A few data were missing in the database, corresponding to different houses for different variables, and thus missing data were considered randomly distributed (up to 8% of missing values for one variable retained in the model). We then used a multiple imputation method implemented in the R package ‘*amelia II*’ to estimate the missing values [38], [39]. Following established guidelines we constructed 5 datasets with imputed data [38], [40]. For each logistic regression, the value of each coefficient was calculated as its mean value across the 5 datasets, and standard errors were calculated taking into account the mean intra-dataset variance and the between-dataset variance [40].

We then selected for further multivariate analysis a sub-set of 29 variables that had *P*-values<0.1 from a likelihood ratio test in the univariate analysis. Because of the redundancy and overlap of several of these variables, as assessed by correlation analysis, these were further reduced to a subset of 9 variables selected to incorporate the maximum independent information in our model while keeping covariation among variables at a minimum (see results and Table S2). We evaluated the goodness of fit of the complete model (i.e. the model with all nine variables) based on a generalised coefficient of determination [41], and the potential for over-dispersion in the data [42].

### Multimodel inference and model averaging

Multivariate analyses were performed using the framework of multimodel inference and selection followed by model averaging [42] to assess the support of each model in terms of Akaike's Information Criterion (AIC). We also determined the odds ratio associated with each of the 9 variables, with their 95% confidence intervals (95%CI) constructed using multi-model estimations of each coefficient's variance [42], [43]. All analysis were performed in Matlab (R2012b, The Mathwork).

We performed 512 logistic regressions including different combination of zero up to nine of the selected variables and determined the likelihood (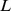) for each of the models. The AIC of each model (corrected for small sample size) was calculated as 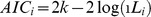 where k is the number of parameters estimated in the model. The final AIC was taken as the mean AIC from the 5 imputed datasets [44]. Models were then ranked from the best supported model (with the minimum value of AIC, AIC_min_) to the least supported one (maximum value of AIC). Akaike differences were calculated as 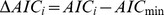 and models with 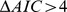 were considered to have a considerably lower support than the best supported model [43].

The Akaike weight (W_AIC_) for each model was defined as: 
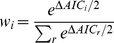
 and it provides the probability for each model to be the best model. The relative importance of a particular variable was then calculated as the sum of the Akaike weights of all models that contained this particular variable.

Finally, to obtain a model including the most complete information and the best predictive ability, we performed model averaging, in which each parameter was weighted by the W_AIC_ of each model and averaged for all 512 models [45]. Thus, for parameter 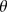 linking infestation with a variable in the logistic regression, the average value over all R models is:
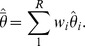



A confidence interval was calculated for each parameter assuming a normal distribution with a total variance associated to the parameter. This variance accounted for (1) the uncertainty in the parameter value given a certain model 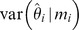, and for (2) the uncertainty associated with model selection, as follows:
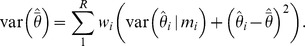



We then derived a confidence interval for the odds ratio associated with each parameter by taking the exponential of the lower and upper bounds of the parameter confidence interval. Finally, we evaluated a posteriori interactions among the six most supported variables of the best model, and constructed 15 models including one pairwise interaction. The statistical significance of each interaction was tested using a Student's *t* test.

### Predicting house infestation from the averaged model

For each household, the probability of infestation was calculated based on the model's averaged parameters to assess the reliability of the model in terms of sensitivity and specificity. We thus calculated the generalized coefficient of determination (following [41]) to assess the fit of the model to observed data. To assess predictions at a coarser level, we also defined 15 groups of 20 houses according to their predicted probability of infestation. Houses from the first group had the 20 lowest predicted probabilities of infestation, and those from the last group had the 20 highest predicted probabilities (due to a sample size of 308, the first and last group actually consisted of 23 and 24 houses). For each group, we calculated the observed infestation probability defined as the proportion of infested houses in the group. The relationship between predicted and observed infestation was evaluated by correlation and regression analysis.

## Results

### The Mayan household

The eco-bio-social characteristics of a total of 308 households were obtained from the field survey. A typical household was composed of a family of 4–5 persons (4.1±0.1), led by a man in 77% of the cases. Most worked as subsistence farmers (38%), some in construction or manufacture (14%) and only 22% had a regular work contract. Sixty-three percent received social welfare benefits (“*Oportunidades*” program). Education level reached primary school for most men and women (63%).

**Figure 1:**
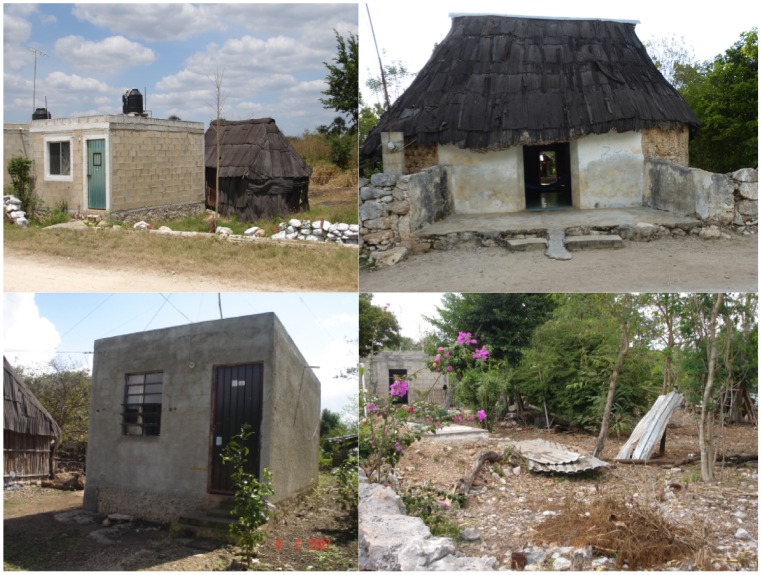
Typical housing and peridomestic structures in rural Yucatan, Mexico.

The houses had been built 20±1 years ago and consisted of 2.1±0.1 adjacent rooms. This included 1.6±0.1 bedrooms and rooms had an average of 1.5±0.1 windows (Fig. 1). Houses were of cement block construction (96%), often fully plastered walls (63%), with cement floors (93%). Similarly, roofs were made of cement/concrete; only 5% were thatched and 8% from tin. Fourteen percent of houses had no sanitation system.

Houses were surrounded by a peridomestic area of on average 1300 m^2^, limited by a fence of piled rocks (84% of houses). Vegetation was scarce close to the house and trees were somewhat denser further from it (>10 m). Many families kept domestic animals all year round (58%), the most frequent being dogs (52%), chickens (49%), cats (34%), and songbirds (14%). Other animals such as rabbits, pigs, sheep, horses and cows were rather rare (<3%). Animals were usually kept close to the house (<10 m, 83% of cases). Songbirds were kept in suspended cages attached to the fronts of houses, chickens and turkeys were sometimes kept in a corrals/coops (22%), while other animals had free range in the peridomicile. Construction materials were sometimes stored in the peridomicile (25%), while corn or other grains (13%) and firewood (10%) were stored inside the house. The peridomicile area was cleaned at least once a week in most cases (66%), usually by men. About 55% of families used domestic insecticide products such as mosquito coils (45%), plug-in repellents (32%) or spray insecticides (55%) on a regular basis, but only a few (5%) resorted to professional insecticide spraying. Thirty-nine percent of households reported having seen triatomines in their house.

### Univariate analysis of infestation

Triatomine transient domestic infestation was detected in 46 of the 308 households (14.9%), corresponding to 10/70 houses (14.3%) in Sudzal, 9/106 houses (8.5%) in Bokoba, and 27/132 houses (20.5%) in Teya. The colonization index was of 5/46 (10.8%) and the density index was of 2.9 triatomines/house, ranging from 1 to a maximum of 43 triatomines/house. All these data were similar to what has been previously observed in these same villages [37], which are also very representative of other villages from the region [23], [24], [46].

The potential association of house transient infestation with eco-bio-social characteristics was first assessed by logistic regressions and 29/127 variables included in the survey were found correlated with house infestation at a *P*<0.1 level (Table S2). Importantly, none of the variables related to the socio-economic status of the household, the education level or general cultural practices such as sleeping or cleaning habits of the house or peridomicile were found to be associated with infestation. The use of various domestic insecticide products or peridomestic pesticides had also no relationship with infestation. Similarly, most variables describing housing and peridomicile structure and organization, including floor, walls or roof type, number of rooms, number of inhabitants, peridomestic vegetation type and density, were not significantly associated with infestation.

On the other hand, the 29 variables associated with transient domestic infestation (Table S2) were related to the presence of specific domestic animals such as dogs, chickens and perching birds, housing condition such as wall plastering, and the surroundings of the houses such as the presence of piles of rocks in the backyard/peridomicile, the proximity of a street light and the location of a house in the periphery of the village.

### Multivariate analysis and modelling

We first further reduced the number of variables because of the redundancy and correlation of several of these, so that we could incorporate the maximum independent information in our model and limit covariation among explanatory variables (Table S3). For example, three variables described the presence of dogs: ‘Presence/absence of dogs’, ‘Number of dogs’ and whether dogs were ‘free ranging, enclosed in a corral or tied on a leash, or absent’. We thus eliminated the first variable because of its complete redundancy with the other two and further selected the variable with the lowest *P*-value in the univariate analysis, which led to retain the variable ‘Number of dogs’ for further modelling. This process resulted in the elimination of 20 variables and only 9 variables were kept for multivariate analysis, with pairwise correlations among them always lower than 0.25 (Table S3). These variables included the number of dogs, presence of chickens in a corral or free-ranging, proximity to the periphery of the village, practice of removing trash from the peridomicile, presence of rock piles close to the house, presence of songbirds, the storage of firewood inside the house, complete wall plastering and the proximity of a public street light. We then evaluated the complete model (i.e. the model with all 9 variables) and found no obvious over-dispersion in the data (Pearson's goodness of fit, 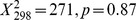, 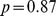, or using the residual deviance: 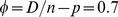). Furthermore, this model accounted for 29% (R^2^) of the variation in infestation.

Analysis of all 512 models including different combinations of the 9 variables indicated rapidly increasing AIC scores and 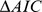, but 10 models presented a ΔAIC of less than 4.5 and were thus considered to receive support from the data (Table 1). These 10 models included the complete model, but the best supported model was comprised of only 8 of the variables and had a coefficient of determination of 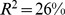. Since all 10 models contained at least 6 variables, infestation by triatomines may not be attributed to a single or few factors, but rather seemed to depend on a complex combination of conditions.

**Table 1:** AIC support of the 10 best models predicting transient house infestation.

Model rank	Variables included in model^a^	AIC	ΔAIC	W_AIC_
1	‘1 2 3 4 5 7 8 9’	226.5	0	0.20
2	‘1 2 3 4 5 7 9’	227.4	0.9	0.12
3	‘1 2 3 4 7 8 9’	228.5	2.0	0.07
**4**	**‘1 2 3 4 5 6 7 8 9’**	**228.9**	**2.4**	**0.06**
5	‘1 2 3 4 5 7 8’	229.2	2.7	0.05
6	‘1 2 3 4 5 6 7 9’	229.8	3.3	0.04
7	‘1 2 3 4 5 7’	229.8	3.3	0.04
8	‘1 2 3 4 6 7 8 9’	230.1	3.6	0.03
9	‘1 2 3 4 7 9’	230.6	4.1	0.02
10	‘1 2 3 4 7 8’	231.0	4.5	0.02

a Variables are coded as follows: 1) Number of dogs, 2) Presence of chickens in coops, 3) Distance to periphery of village, 4) Cleaning trash from peridomicile, 5) Presence of rock piles away from the house, 6) Presence of pet perching birds, 7) Storage of firewood inside the house, 8) Complete plastering of walls, 9) Distance to public street lights. Bold fonts indicate the complete model with all 9 variables.

We then proceeded to model averaging to identify the strongest determinants for house infestation and evaluate the predictive power of our model. The Akaike weight of each variable in the averaged model indicated that five variables could be considered of high importance in defining house infestation with W_AIC_>0.9, two additional variables were of secondary importance with 0.7<W_AIC_<0.9, and the remaining had limited contributions (Table 2). As reported before [35], the location of a house at the periphery of a village increased the risk of infestation and had a very high weight. Keeping chickens in a coop or corral was a major determinant of infestation - it resulted in a 2.4 fold higher risk of infestation by triatomines - while having free-ranging chickens had no effect (Table 2). Cleaning trash from the peridomicile area similarly doubled the risk of infestation, and the presence of more than two dogs also significantly increased this risk. The storage of firewood inside the house was a major protective factor in the averaged model, although the individual effect was not statistically significant. Risk factors of secondary importance consisted of the proximity of a public street light, which had been identified before [37], and the presence of rock piles in the peridomicile. Again, the individual contributions of these factors in terms of odds ratio was not significant. Finally, the complete plastering of walls and the presence of perching birds had very minor weights in the model and may thus be of limited relevance as determinants for infestation. Potential interactions between the six most supported variables were further evaluated a posteriori, but none of them reached statistical significance.

**Table 2:** Key eco-bio-social determinants for house infestation by non-domiciliated *Triatoma dimidiata*.

Variable	WAIC	Sample size, level	Odds ratio [95% CI]
Distance to periphery of village	0.95#	298	0.56 [0.36–0.88]*
Storage of firewood inside the house	0.94#	249 No	-
		29 Yes	0.12 [0.01–1.57]
Presence of chickens	0.93#	220 None	-
		42 In a coop	2.39 [1.01–5.66]*
		46 Free-ranging	0.39 [0.12–1.30]
Cleaning trash from peridomestic areas	0.93#	114 No	-
		168 Yes	2.68 [1.19–6.04]*
Number of dogs	0.92#	308	1.39 [1.07–1.81]*
Distance to public street light	0.78	308	0.08 [0.004–1.88]
Presence of rock piles away from the house	0.73	230 No	-
		78 Yes	1.80 [0.83–3.89]
Complete plastering of walls	0.64	82 No	-
		221 Yes	0.63 [0.31–1.32]
Presence of perching birds	0.27	265 None	-
		15 Sometimes	1.22 [0.46–3.25]
		27 All year round	1.21 [0.57–2.58]

# indicates the most important variables in the averaged model and

* indicates a statistically significant odds ratio of variables when considered individually.

### Predicting house infestation from risk factors

**Figure 2:**
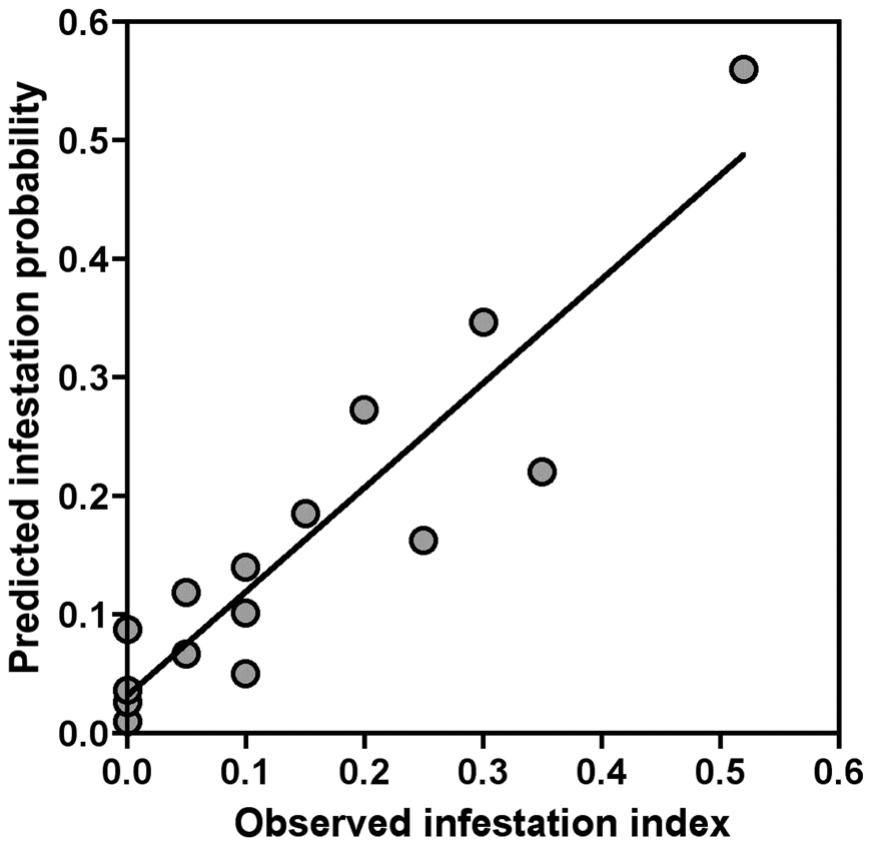
Comparison of predicted infestation probability and observed infestation in groups of 20 houses based on the averaged model. Houses were divided into 15 groups based on their predicted individual probability of infestation and their mean probability of infestation was compared with the observed infestation index for each group. The relationship was: Predicted infestation = 0.032+0.878*Observed infestation (R^2^ = 0.85, *P*<0.0001).

We then tested if these determinants of infestation could be used to predict house infestation, and thus be used to target potential vector control interventions. The generalized coefficient of determination of the averaged model was 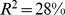, indicating that about a third of the variance in the observed pattern of infestation at the level of a single house could be predicted by the model. The model allowed the correct identification of 90% of non-infested houses (specificity), while 41% of infested houses could be correctly identified (sensitivity). We also grouped houses according to their predicted probability of infestation to assess the reliability of the model at a somewhat larger scale (groups of 20 houses) by comparing the predictions with the observed infestation index of each group of houses. In this case, the averaged model provided an excellent prediction of infestation probability compared with the observed infestation index (R^2^ = 0.85, *P*<0.0001, slope = 0.878) and was thus able to explain most of the variance in the infestation pattern (Fig. 2).

## Discussion

Transient house infestation by non-domiciliated triatomine vectors remains a key challenge for the design of sustainable vector control interventions and further reduction of the burden of Chagas disease [47]. This infestation pattern makes conventional insecticide spraying poorly effective, and a better understanding of the determinants of infestation is needed to formulate novel vector control strategies [31], [32], [36], [48]. In fact, risk factors for house infestation by these non-domiciliated triatomines remain poorly understood, limiting the breadth of potential control interventions to be tested. We performed here the first detailed analysis aimed at identifying possible determinants of domestic infestation by *T. dimidiata*.

From the initial univariate screening of 127 eco-bio-social variables describing the rural ecosystem, it was clear that variables associated with infestation by invasive triatomines are distinct from the determinants usually associated with infestation and colonization with domiciliated triatomines. Indeed, socio-economic status or housing quality were clearly not relevant for infestation [23], [24]. Similarly, even indoor use of a variety of domestic insecticide products was found irrelevant to prevent triatomine infestation. On the other hand, the strongest five determinants for infestation that were identified through our model selection and averaging approach included the number of dogs, having chickens in a corral, the practice of cleaning of trash from the peridomicile, and being located in the periphery of the village, which all favoured infestation, while the presence of firewood inside houses appeared protective. To a lesser extent, the proximity of public lights and the presence of rock piles in the peridomiciles were also associated with infestation according to our model. The relationship between the storage of firewood and the cleaning of the peridomicile with house infestation is difficult to interpret. Indeed, the presence of firewood has usually been associated with increased infestation risk [49] due to the passive transport of bugs and the potential refuge it provides. Alternatively, households may use smoke from firewood to repel insects from their house, as reported in the state of Chiapas, Mexico [50]. Peridomicile cleaning would also be expected to limit peridomestic infestation, and as a consequence the dispersal of peridomestic bugs towards houses. Studies on peridomicile management aimed at eliminating peridomestic bug colonies suggest it may indeed contribute to integrated vector control [32], [51]. Alternatively, peridomicile cleaning may reduce the availability of refuges and increase bug dispersal, and as a consequence favour domestic infestation. This potential effect of environmental management has not been considered yet, and may have contributed to the pattern observed in this study.

On the other hand, all other determinants for transient infestation that we identified here are consistent with our previous hypothesis that poorly-fed bugs from both the peridomicile and surrounding sylvatic areas, foraging for blood sources, are infesting houses [26], [52]. Thus, houses located in the periphery would be more at risk of infestation as shown before [34], [35], [52]. The presence of dogs and chickens, the most common domestic animals, would be attractive food sources that may be effectively detected by foraging bugs [53]. Further studies of *T. cruzi* infection in dogs may provide additional information on their role as domestic reservoirs in the villages. Interestingly, only chickens held in a corral or coop seem to contribute to infestation, while free-ranging chickens do not play a role. Thus, a concentrated and captive chicken population may provide a stronger signal to attract bugs and an easier food source. The contribution of dogs and chickens in infestation by *T. infestans* has been observed previously [11], [54], [55]. Public street lights may then interfere with the dispersal process and attract bugs to nearby houses as suggested before [37] and rock piles may provide additional peridomestic refuges for bugs.

The reliability of our model was further assessed by evaluating its ability to predict house transient infestation based on the determinants identified. At the level of an individual house, our averaged model was able to account for about 30% of the presence/absence of infestation by *T. dimidiata*. However, when houses were grouped according to their predicted infestation, the model then accounted for up to 85% of the variations in the observed infestation index. Unfortunately, the predictive value of models is rarely reported by authors attempting to identify risk factors, even though they often base subsequent vector control interventions on such studies, with little certainty that infestation will actually be affected [9], [12]–[15], [17]–[19]. The general practice is to use odds ratio statistics to identify key risk factors, but the actual capacity of the key factors to predict the level of risk is not assessed. This lack of assessment occurs with risk studies of other vector borne diseases as well, including malaria or dengue [56]–[60]. To our knowledge such evaluation has been attempted only once from a multimodel inference approach similar to the one adopted in this paper. This study identified a limited number of risk factors contributing to the infestation of domiciles or of chicken coops by *T. infestans* in the Grand Chaco, Argentina [54] and the authors genuinely tested the predictive capacity at the site level of their best statistical model. They reported levels of sensitivity and specificity of 49% and 82% in domicile infestation, and 65% and 71% in chicken coops infestation, which is similar to the sensitivity (41%) and specificity (90%) we reported. In our study, despite the fact that bug dispersal is a complex dynamic process with potentially many interacting factors, which can lead to very variable infestation outcomes, the few variables we identified account for most of the variability in terms of infestation at the level of groups of houses (R^2^ = 0.85). These data suggest that we have identified the key determinants of infestation in this model and that additional determinants we may have missed will be minor contributors to infestation. While the model was not tested on additional villages, the representativeness of the studied villages suggests that our findings may be extrapolated to villages with similar characteristics. Additional studies should help explore further generalization of our results. Thus, infestation is actually associated with a rather limited set of risk factors, each having a modest contribution, and their particular combinations and synergism is what seems to be associated with infestation of specific houses. Importantly, our results suggest that targeting a single risk factor may be ineffective for vector control and that combined integrated interventions targeting multiple variables may be required to adequately reduce infestation by *T. dimidiata*. Nonetheless, the small number of factors to be modified suggests that such integrated control might be feasible.

In conclusion, our search for eco-bio-social determinants for house transient infestation by non-domiciliated *T. dimidiata* clearly identified several factors, including the presence of dogs and chickens in the peridomicile, potential refuges such as rock piles, and the location of the house close to the vegetation at the periphery of the village, and proximity to public street lights. These factors allowed us to explain most of the variation in infestation within villages in rural Yucatan, Mexico. These results may be used for risk stratification within villages for both vector surveillance and control. They also suggest that combined integrated vector interventions, informed by an Ecohealth perspective, should target several of these factors to effectively reduce infestation and provide sustainable vector control.

## Supporting Information

Table S1 **List of studied variables.**
(XLSX)

Table S2 **List of significant variables from the univariate analysis.**
(DOCX)

Table S3 **Correlation analysis among the 9 variables retained in the multivariate analysis.**
(XLSX)
