# Supplementary material for: Eco-Bio-Social Determinants for House Infestation by Non-domiciliated Triatoma dimidiata in the Yucatan Peninsula, Mexico
Source: PLoS Negl Trop Dis. 2013 Sep 26;7(9):e2466. doi: 10.1371/journal.pntd.0002466 (PMC3784500; doi:10.1371/journal.pntd.0002466)
Supplement: Table S2 — List of significant variables from the univariate analysis. (DOCX) [file pntd.0002466.s002.docx]

**Supplementary Table S2. List of significant varariables from the univariate analysis.**

|  | **LRp** | **Variable** | **b (coefficient)** | | | **Wald *P* value** | | |
| --- | --- | --- | --- | --- | --- | --- | --- | --- |
| 1 | 2E-04 | Presence of avian corral (no, yes) | -2.1172 | 1.2843 |  | 0 | 0.0002 |  |
| 2 | 9E-04 | Presence of chicken coop (no, yes) | -2.0569 | 1.186 |  | 0 | 0.0006 |  |
| 3 | 9E-04 | Keeping of birds (in a corral, free, no birds) | -2.0069 | 1.3783 | -0.2123 | 0 | 0.0002 | 0.6812 |
| **4** | **0.001** | **Number of dogs** | **-2.1648** | **0.3772** |  | **0** | **0.0008** |  |
| **5** | **0.004** | **Keeping of chickens (in coop, free-ranging or no chicken)** | **-1.9253** | **1.2321** | **-0.4261** | **0** | **0.0014** | **0.4476** |
| **6** | **0.005** | **Distance to periphery of village (in meter)** | **-1.0081** | **-0.0054** |  | **0.0004** | **0.0077** |  |
| 7 | 0.007 | Presence of cage for perching birds (no, yes) | -1.9247 | 1.1137 |  | 0 | 0.0045 |  |
| **8** | **0.007** | **Cleaning trash from patio (no, yes)** | **-2.4567** | **1.0098** |  | **0** | **0.0114** |  |
| 9 | 0.01 | Number of birds | -2.074 | 0.84 |  | 0 | 0.0096 |  |
| 10 | 0.013 | Presence of animals (no, yes) | -2.3206 | 0.8755 |  | 0 | 0.0181 |  |
| 11 | 0.017 | Presence of perching birds (no, yes) | -1.9161 | 0.967 |  | 0 | 0.0123 |  |
| 12 | 0.022 | Presence of dogs (no, yes) | -2.1823 | 0.7565 |  | 0 | 0.0252 |  |
| **13** | **0.025** | **Presence of rock piles >10 m from the house (no, yes)** | **-1.9761** | **0.7721** |  | **0** | **0.0216** |  |
| 14 | 0.031 | Location of birds (no birds, <10 meters from the house, > 10 m) | -2.0794 | 0.9808 | 0.8267 | 0 | 0.0808 | 0.0172 |
| **15** | **0.032** | **Presence of perching birds (no birds, sometimes, all year round)** | **-1.9161** | **0.5298** | **1.2229** | **0** | **0.4299** | **0.0063** |
| 16 | 0.038 | Presence of chickens (no, yes) | -1.9837 | 0.6889 |  | 0 | 0.0356 |  |
| **17** | **0.041** | **Presence of firewood inside the house (no, yes)** | **-1.6835** | **-1.6487** |  | **0** | **0.1103** |  |
| 18 | 0.045 | Keeping of dogs (no dogs, enclosed or tied, free-ranging) | -2.1972 | 0.5878 | 0.8644 | 0 | 0.2355 | 0.0152 |
| 19 | 0.051 | Location of perching birds (no birds, in the patio, inside the house) | -1.9161 | 0.86 | 1.2229 | 0 | 0.0558 | 0.0558 |
| **20** | **0.052** | **Complete plastering of wall (no, yes)** | **-1.2685** | **-0.662** |  | **0** | **0.048** |  |
| 21 | 0.056 | Time since last cleaning of the chicken coop | -1.8718 | 0.619 | -0.8362 | 0 | 0.0689 | 0.2712 |
| **22** | **0.056** | **Distance to public street light** | **-1.1973** | **-0.0266** |  | **0.0003** | **0.0805** |  |
| 23 | 0.061 | Keeping of poultry (no poultry, enclosed, free-ranging) | -1.9315 | 0.6931 | -0.2841 | 0 | 0.047 | 0.5637 |
| 24 | 0.068 | Presence of dogs (no dogs, sometimes, all year round) | -2.1823 | 0.796 | 0.7583 | 0 | 0.1621 | 0.0299 |
| 25 | 0.073 | Corral for poultry other than chickens (no, yes) | -1.8036 | 1.244 |  | 0 | 0.0551 |  |
| 26 | 0.074 | Location of chickens (no chickens, <10m from the house, >10m) | -1.9889 | 1.1135 | 0.6026 | 0 | 0.0515 | 0.0909 |
| 27 | 0.075 | Presence of poultry other than chickens (no, yes) | -1.8245 | 0.9773 |  | 0 | 0.0587 |  |
| 28 | 0.086 | Number of poultry | -1.9051 | 0.0348 |  | 0 | 0.0728 |  |
| 29 | 0.1 | Presence of thatched construction near the house (no, yes <10m away) | -1.86 | 0.3074 |  | 0 | 0.089 |  |

Gray shaded rows with bold fonts indicate the variables that were included into the multivariate analysis. *: *P*-value associated with a Likelihood Ratio test.
